# Imported visceral leishmaniasis and immunosuppression in seven Norwegian patients

**DOI:** 10.1186/s40794-019-0092-x

**Published:** 2019-08-22

**Authors:** Thomas Schwartz, Mogens Jensenius, Bjørn Blomberg, Cathrine Fladeby, Arild Mæland, Frank O. Pettersen

**Affiliations:** 1 Department of Infectious Diseases, Ullevål, Oslo University Hospital, Oslo, Norway; 2 Department of Medical Microbiology, Vestre Viken, Drammen, Norway; 3 Norwegian National Advisory Unit on Tropical Infectious Diseases. Department of Medicine, Haukeland University Hospital, Bergen, Norway; 4 Department of Clinical Science, University of Bergen, Bergen, Norway; 5 Department of Microbiology, Ullevål, Oslo University Hospital, Oslo, Norway; 6 Regional Advisory Unit of Imported and Tropical Diseases, Oslo University Hospital, Oslo, Norway

**Keywords:** Leishmaniasis, Travel medicine, Rheumatic disease, Autoimmune disease, Immunosuppression, Tumor necrosis factor alpha inhibitor, Methotrexate

## Abstract

**Background:**

Visceral leishmaniasis (VL) is a protozoal disease that may be aggravated by immunosuppression. In recent years, a growing number of patients with chronic diseases use biological treatment. When such immunosuppressed patients travel to endemic areas, they are facing the risk of VL. Increased incidence of leishmaniasis is reported in endemic areas like the Mediterranean basin, an area frequently visited by Norwegian tourists. This may lead to an increased number of patients, many presenting to health personnel unfamiliar with the disease, in their home countries.

**Methods:**

We reviewed the files of seven immunosuppressed patients with VL, admitted to Oslo and Haukeland University Hospitals in Norway in the period 2009–2018.

**Results:**

The patients were 41–83 (median 66) years of age; four had rheumatic disease all of whom used methotrexate; one had advanced HIV infection, one had inflammatory bowel disease and one had myelofibrosis. *Leishmania infantum* was confirmed in five patients by polymerase chain reaction (PCR) and sequencing. In the remaining two patients, a definite *Leishmania* species could not be identified. All patients had a history of recent recreational travel to Spain. Most patients underwent extensive diagnostic work-up before diagnosed with VL. All received treatment with liposomal amphotericin B and all were cured; albeit two after re-treatment due to relapse.

**Conclusions:**

Visceral leishmaniasis is a potentially life-threatening but usually treatable condition. It is endemic in Southern Europe, including popular tourist destinations such as the Mediterranean basin. It is relatively unknown to most medical practitioners in non-endemic areas and clinical vigilance is required to identify those who are infected.

## Background

Leishmaniasis is a chronic protozoal disease, caused by parasites of the genus *Leishmania* and is transmitted by sandflies, which are active at dusk and night and even indoor. Visceral leishmaniasis (VL) is the most severe form with 95% mortality untreated [1]. It is caused either by *Leishmania donovani* which is transmitted between humans, or by *Leishmania infantum* which is a zoonosis and known as *L.chagasi* in the New World. The disease can present years after transmission [1]. VL has caused devastating epidemics during famine and wartime, particularly on the Horn of Africa [2]. Ninety percent of the cases occur on the Indian subcontinent, the Horn of Africa (*L. donovani*) and in Brazil (*L. infantum*) [1, 3]. VL is also endemic to Southern Europe, including popular tourist destinations lining the Mediterranean basin, however to a smaller extent. In the period 2006–2014, the reported average number of VL cases per year in Spain and France were 206 and 14, respectively (WHO, Global Health Observatory). In this region, the responsible parasite is *L. infantum* and dogs act as the primary zoonotic reservoir [4]. The incidence of VL appears to increase in Southern Europe [5]. Acre, et al., recently documented the largest leishmaniasis outbreak in Europe in modern times, occurring outside of Madrid in the years 2009–2012 with 446 reported cases [6]. HIV-induced immunosuppression increases the risk of developing VL once infected [7, 8]. Among 160 patients with VL in the Madrid outbreak, 16 had HIV infection and 34 were immunosuppressed due to other causes. In recent years, the use of biological treatment for various chronic diseases has increased dramatically, improving many people’s lives. Thus, they take part in the modern international mass tourism, including areas endemic to VL. The number of susceptible patients facing the risk of VL has therefore increased. Here, we present seven immunosuppressed patients diagnosed with imported VL in Norway.

## Methods

From 2009 to 2018, our referral hospitals diagnosed seven immunosuppressed patients with VL after returning from travel to Spain. Five patients were diagnosed and had treatment initiated at Oslo University Hospital (OUS) and one (patient 6) at Haukeland University Hospital (HUH) in Bergen. Patient 2 fell sick in Thailand where he was diagnosed and treated before he was transferred to OUS for treatment completion. All patients gave their oral and written consent to the use of their data in this report. Our article is a retrospective descriptive case series without interventions. Approval from Regional Ethics Committee for Medical Research was therefore not relevant. Publication has been done in accordance with OUS and HUH’s policy of personal and private security.

**Fig. 1:**
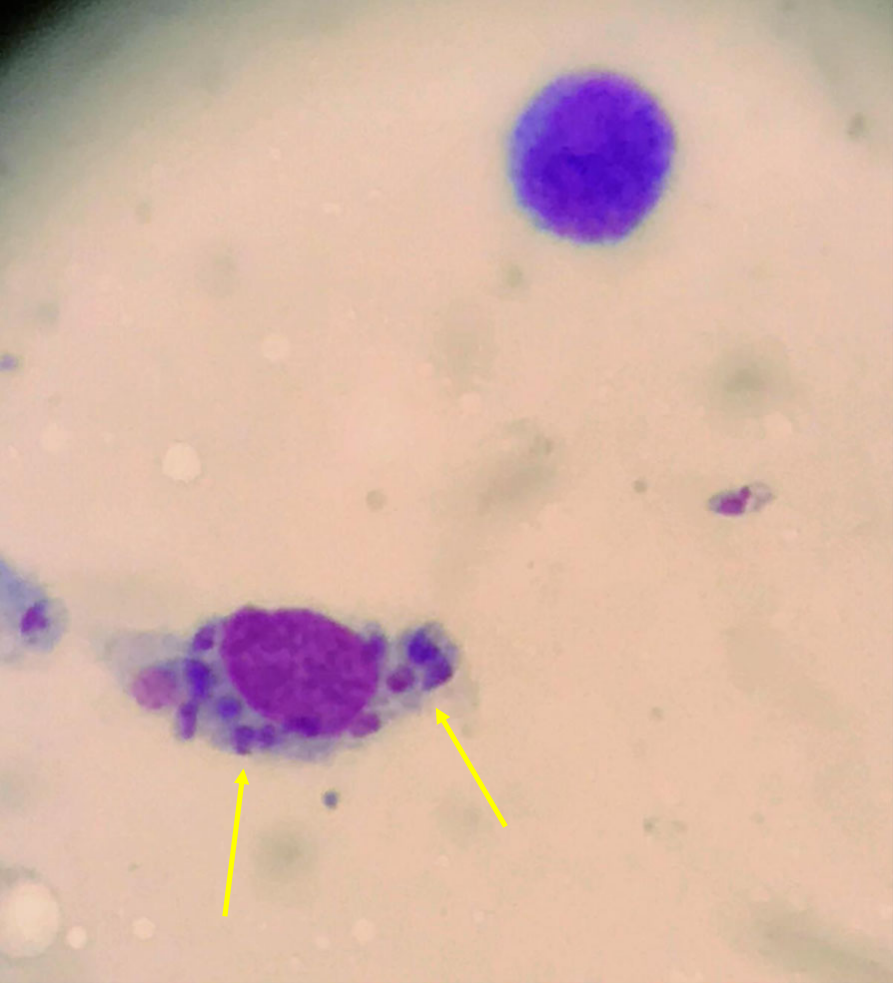
Amastigotes (yellow arrows) in bone marrow aspirate, with May-Grünwald-Giemsa staining from patient 5, magnified × 40

The VL diagnosis was based on a combination of histopathological, serological, polymerase chain reaction (PCR) and sequencing analysis. Pathologists at our university clinics performed histological analysis (Fig. 1). Leishmania serology tests were analyzed at the Public Health Agency of Sweden, Stockholm, Sweden. The serological test can identify *Leishmania*-infection but cannot distinguish between species. Since 2015, Department of Microbiology, OUS, has offered an in-house real-time PCR using18S rRNA gene as target for *Leishmania* detection and *Leishmania hsp70* gene sequencing for *Leishmania* species identification [9].

## Results

Table 1 shows the epidemiological characteristics of the seven patients (median age 66 years, range 41–83 years). Six patients (85,7%) were males, four (57,1%) had rheumatic disease, one had advanced HIV infection, one inflammatory bowel disease (ulcerative colitis) and one myelofibrosis. All patients presented with the triad of fever, pancytopenia and splenomegaly. Comprehensive diagnostic procedures such as computer tomography, bone marrow examination, microbiological tests and culturing were performed in all patients due to the severity of the clinical presentations. Patient 5 was immunosuppressed due to an unrecognised HIV infection with a CD4 cell count of 19 cells/mm^3^ at presentation. All, except patient 5, acquired infection during recreational travels.

**Table 1:** Epidemiological characteristics of seven immunosuppressed patients with visceral leishmaniasis, 2009–2018, Norway

Case no	Sex	Age	Chronic underlying disease	Immunosuppressive treatment	Fever, splenomegaly, weight loss	Other clinical features and duration of symptoms at diagnosis	Travel history	Likely place of transmission
Patient 1	M	66	Rheumatoid arthritis,	MTX 17.5 mg/week for 7 years and adalimumab	Yes	6 months of constitutional symptoms and oesophageal candidiasis. A chronic skin ulcer acquired in Spain one year ago was diagnosed as CL. Then, a prednisolone course due to worsening of RA was followed by the clinical picture of VL	Recreational travel to Spain > one year ago	Spain
Patient 2	M	78	Psoriasis arthritis	MTX 20 mg/week for > 5 years and prednisolone	Yes	Weight loss over a year. While in Thailand, fever and asthenia developed hospitalization. Splenectomy was performed after 10 days in hospital due to intractable pancytopenia	Several recreational travels to Thailand the last year; the last five years to Spain. Professional travels to Pakistan, East Africa and India the last 20 years	Unknown
Patient 3	M	79	Rheumatoid arthritis	MTX12.5 mg/week for several years and prednisolone	Yes	6 weeks of constitutional symptoms, prior to VL diagnosis. 16 months after treatment, he fell sick from a testis B-cell lymphoma. He then had a relapse of VL, prior to cytostatic treatment	Recreational travels to Spain one year, to Turkey 8 years and to Italy, Spain, Portugal some 20 years ago, respectively	Unknown
Patient 4	M	57	Myelofibrosis	No	Yes	Salmonella gastroenteritis while in Tanzania. Admitted 2 months later with constitutional symptoms and increasing weight loss 5–15 kg.	Holiday house in Spain. Recreational travel to Tanzania 2 months ago	Spain
Patient 5	M	41	HIV	No	Yes	Constitutional symptoms and 15 kg weight loss the last two months. On admission he had oesophageal candidiasis; HIV and VL were diagnosed respectively. Relapse of VL after 3 months, CD4, 119/ml	Prior to 2010 he lived in Portugal. He then lived in South Africa 2010–12, in Cape Verde 2012–14; thereafter in Norway. Many recreational and profession travels in Spain.	Spain, Portugal
Patient 6	M	83	Rheumatoid arthritis	MTX 20 mg/week for 10 months, initially together with prednisolone, but last 6 months MTX alone	Yes	Admitted with constitutional symptoms, two weeks after returning from Spain. Extensive diagnostic work-up and progressive weight loss 4-13 kg until diagnosed VL	Recreational travel to Spain	Spain
Patient 7	F	46	Ulcerative colitis	Azathioprine and infliximab for 1.5 years – discontinued 6 and 8 months after symptoms respectively	Yes	Splenectomy on the suspicion of lymphoma	Recreational travels to Spain	Spain

Investigations, treatment and outcome of the patients are listed in Table 2. In five of seven patients (71,4%, patient 1, 4, 5, 6 and 7), PCR and sequencing were performed on bone marrow, spleen aspirate, spleen biopsy and/or peripheral blood and *L. infantum* infection was confirmed. In one case only, the initial diagnosis was made by PCR (patient 7). In five cases (71,4%), the initial leishmaniasis diagnosis was based on direct microscopic detection of amastigotes within macrophages in aspirate (Fig. 1), either from skin (patient 1), spleen (patient 2 and 6) or bone marrow (patient 3 and 5), before serology or PCR was performed.

**Table 2:** Investigations, treatment and outcome in seven immunosuppressed patients with visceral leishmaniasis, 2009–2018, Norway

Case no	Sex	Age	Microscopy (site)	Serology	PCR (site)	Sequencing	Hemoglobin (13.4–17.0 g/dl)	Leukocyte (3.5–10.0 10^9^/L)	Thrombocytes (145–390 10^9^/L)	Treatment – total cumulative doses of AmB/L-AmB	Outcome
Patient 1	M	66	Positive (skin ulcer)	Positive	Positive (bone marrow)	*L.infantum*	8.8	1.4	165	2350 mg(26 mg/kg)	Cured
Patient 2	M	78	Positive (spleen)	Positive	NA	NA	9.3	1.0	20	1740 mg^a^(23 mg/kg)	Cured
Patient 3	M	79	Positive (bone marrow)	Positive	NA	NA	8.6	1.0	15	1800 mg(20 mg/kg)	Cured. Relapse after 17 months, recovery after re-treatment L-AmB 2000 mg. Died two years later of unknown cause
Patient 4	M	57	Positive (spleen aspirate)	Positive	Positive (blood)	*L.infantum*	7.6	0.9	105	1400 mg21 mg/kg	Cured
Patient 5	M	41	Positive (bone marrow)	NA	Positive (blood)	*L.infantum*	8.5	1.8	95	2040 mg (29 mg/kg)	Cured. Relapse after 3 months, recovery after re-treatment L-AmB 1400 mg. FU = 19 months
Patient 6	M	83	Positive (spleen aspirate)	Positive	Positive (spleen and blood)	*L.infantum*	10.0	1.6	110	3300 mg (37 mg/kg)	Cured
Patient 7	F	46	No parasites seen (spleen aspirate)	NA	Positive (spleen)	*L.infantum*	9.8	1.3	61	1760 mg (21 mg/kg)	Cured

Reference values for blood tests are given in brackets. Abbreviations: *M* male, *F* female, *VL* visceral leishmaniasis, *RA* rheumatoid arthritis, *CL* cutan leishmaniasis, *MTX* methotrexate, *NA* not analyzed, *PCR* polymerase chain reaction, *FU* time of follow-up, *dl* deciliters, *L* liters, *BM* bone marrow, *AmB* amphotericin B, *L-AmB* liposomal amphotericin B. ^a^In Thailand: AmB 340 mg + In Norway: 1400 mg L-AmB

Patient 2 and 3 were diagnosed by serology and histology before PCR was available to us. They had both travelled to destinations endemic to other species than *L. infantum* (Table 2), and to the Mediterranean area; therefore a definite *Leishmania* species could not identified.

All four patients with rheumatic disease used methotrexate (57,2% of all patients), either with prednisolone (42,9%), with adalimumab (14,3%) or alone (14,3%). All patients received treatment with liposomal amphotericin B (L-AmB) in total doses 1400 mg–2350 mg (20–37 mg/kg) iv, and all were cured. When diagnosed with B-cell lymphoma 16 months’ post-treatment, patient 3 relapsed with VL without any possible re-exposure to VL. Treatment outcome was not assessed by *Leishmania*-PCR in blood, as this was not available in Norway at that time. Patient 5 relapsed three months after initial treatment; he had not received secondary prophylaxis with L-AmB and was still severely immunosuppressed by HIV. 12 months after re-treatment, there was no sign of VL. Patient 3 and patient 5 initially received total doses of 1800 and 2040 mg with L-AmB, respectively.

## Discussion

During the last decades, the growing population of immunocompromised patients has taken part in modern tourism. They visit areas endemic to leishmaniasis such as southern parts of Europe where they may be exposed to VL. Physicians in non-endemic areas of Northern Europe may not be familiar with VL, the potential risk of infection, or the presence of VL in popular tourist destinations along the Mediterranean. Therefore, we expect an increase in VL-cases in areas non-endemic to *Leishmania* because of the travel activity of immunosuppressed patients to VL endemic areas [6]. Furthermore, the incidence of leishmaniasis cases is increasing in popular tourist destinations such as Spain. Lastly, more cases of VL may be detected due to an improvement of available diagnostic tools such as PCR and sequencing, given that medical doctors in non-endemic areas are alert and primed for the possibility of VL [10–12].

VL has been associated with immunosuppressive disorders like HIV infection, myeloproliferative disorders and cancers, but also with immunosuppressive therapy for autoimmune diseases and after solid-organ transplantation [13, 14]. It is possible that autoimmune diseases per se represent a risk factor for developing VL. But to our knowledge, no systematic studies have addressed the risk of VL in patients with rheumatic or other autoimmune diseases not on treatment with immunosuppressive drugs. In our material, all patients with rheumatic disease (four) received immunosuppressive therapy. VL has been demonstrated in case reports of rheumatic patients treated with tumor necrosis factor alpha (TNF-α) antagonists [15–17] or methotrexate [18–20] and in three non-rheumatic patients on corticosteroids [21]. Patient 7 with ulcerative colitis developed VL while on TNF-α antagonist treatment. In literature, we have only found one single case of VL in a patient with inflammatory bowel disease (Crohn’s disease) on TNF-α antagonists [22].

*TNF-α monoclonal antibodies* have become a cornerstone in modern treatment of many chronic inflammatory disorders and have received an increased attention as a risk factor for VL than other immunosuppressive drugs [17, 23]. TNF-α has pro-inflammatory effects but also plays an important role in the defense against intracellular infections, such as leishmaniasis. Inhibition or lack of TNF-α activity seem to induce an increased risk of leishmaniasis as demonstrated in mouse models [24].

The risk of VL if treated with *methotrexate* is only scarcely documented [18–20]. The patients with rheumatic diseases in our report were all treated with methotrexate. Methotrexate has been used in treatment of rheumatoid arthritis (RA) since the 1960’s and is often combined with other drugs. Notably, in a literature search, Chen, et al., described eight patients with RA developing VL while treated with the combination of adalimumab and methotrexate [23]. Methotrexate is a competitive inhibitor of folic acid-dependent enzymes, leading to impaired purine and pyrimidine synthesis which inhibits proliferation of lymphocytes [25]. As the cellular immune system is important against intracellular infections, it is plausible that this renders patients on methotrexate at risk of VL.

*Corticosteroids* are also widely used in the treatment of autoimmune diseases and, like methotrexate, are often used in combination with other drugs. We have not identified any publications on VL in rheumatic patients on prednisolone alone, although several of the reported cases on anti- TNF-α and methotrexate therapy had received corticosteroids as well [18, 23]. In a large controlled study on 25,139 solid organ transplanted patients in Spain and Brazil, 36 cases of VL was identified [26]. A multivariate logistic regression analysis was performed, and the use of corticosteroids – not mycophenolate, cyclosporine or tacrolimus - was the only risk factor associated with VL. It has also been shown that prolonged use of corticosteroids in *Leishmania*-infected mice, led to decreasing levels of TNF-α and other cytokines, along with increasing numbers of amastigotes in the spleen [27].

L-AmB is the treatment of choice against infections caused by *Leishmania infantum* [28]. Recent guidelines recommend an increased dose of L-AmB in immunocompromised patients: 4 mg/kg/day iv on days 1–5, 10, 17, 24, 31 and 38 (total dose of 40 mg/kg) [28]. Secondary prophylaxis is only recommended to HIV infected individuals as long as CD4 T lymphocytes counts are < 200 cells/mm^3^. Assessment of *Leishmania* PCR to avoid post-treatment relapse is recommended for at least one-year post-treatment [28]. Our patients received total doses of L-AmB in the range of 20-37 mg/kg (Table), i.e. lower than current guidelines. Patient 2 was diagnosed with VL and had treatment initiated in Thailand with non-liposomal amphotericin, before completing therapy in Norway with L-AmB. As we observed two relapses among seven patients after receiving standard doses of L-AmB, it appears reasonable from our limited experience to provide immunocompromised patients with a higher cumulative dose of L-AmB.

Four of the patients had traveled to various destinations endemic to VL, such as Eastern and Western Mediterranean Basin, East Africa, Pakistan and Thailand. It is possible that some of these patients got infected with leishmaniasis years ago, remained asymptomatic, and presented with VL once given immunosuppressive therapy. However, Spain seems like the most probable place of transmission, due to the timing, lengths and/or frequencies of stay in this region, with a possible exception of patient 2 whose place of transmission remains unknown.

All our seven patients underwent a rather extensive diagnostic work-up before a final diagnosis was made. Malignancies and haematological diseases were invariably high on the list of the differential diagnoses. PCR was performed only to *confirm* the diagnosis after positive microscopy, serology or histology, and for species identification in most cases. This could reflect that PCR as a readily available test modality for leishmaniasis is yet to become known to Norwegian medical doctors. Our laboratory at OUS now offers PCR and sequencing (the only in Norway, population 5.25 million people). As a result of this, the awareness of leishmaniasis seems to have increased. During the last three years a total of 32 cases were diagnosed, among whom eight had VL (including one of the present patients) [29].

## Conclusion

We present seven immunosuppressed Norwegian patients suffering from life-threatening VL most likely acquired around the Mediterranean basin. A growing number of people susceptible to VL travel to *Leishmania*-endemic destinations. Medical practitioners, particularly those who care for immunocompromised patients of any cause, need to be aware of the risk of VL and question their patients of their travels including the recreational trip to Spain.

## Data Availability

Not applicable

## Abbreviations

AmB: Amphotericin B
HUH: Haukeland University Hospital
OUS: Oslo University Hospital
PCR: Polymerase chain reaction
RA: Rheumatoid arthritis
TNF-α: Tumor necrosis factor alpha
VL: Visceral Leishmaniasis
